# Gluteal Abscess Secondary to Rectal Perforation Due to Ingested Foreign Body: A Report of Two Cases

**DOI:** 10.7759/cureus.30745

**Published:** 2022-10-27

**Authors:** Emad Amirhom, Joshua S Braganza, Vinayak Gowda, Ajay Shah

**Affiliations:** 1 General Surgery, BronxCare Health System, New York City, USA; 2 General Surgery, WellSpan York Hospital, York, USA

**Keywords:** gastrointestinal tract, incision and drainage, rectal perforation, foreign body, gluteal abscess

## Abstract

Gastrointestinal abscesses are commonly caused by infection and inflammatory and, in rare cases, malignant bowel conditions. This paper reports two cases of rectum/gluteal abscesses due to an ingested foreign body. The goal of this case report is to highlight the need to raise suspicion of foreign body ingestion in the setting of a gluteal abscess with a foreign body that may have caused rectal perforation and subsequent gluteal abscess formation.

## Introduction

There are multiple reasons why an anorectal abscess develops and may even end up causing a fistula if the anal abscess ruptures or with incision and drainage, and thus an epithelialized track forms that connects the abscess at the anus or rectum with the perianal/ perirectal skin [1,2]. The usual causes of anal abscesses are infection of the anal crypt glands, intramuscular injection in the gluteal region, trauma with infection of the same region, or also bacteria entering the root of a hair on the skin. In rare cases, radiation proctitis and malignancy in the form of anal squamous cell carcinoma can present as anal abscesses or even fistulas. Rectally placed foreign bodies can cause mucosal injury causing an abscess and fistula creation [3]. However, what is extremely rare, and we are discussing in this case report, is the possibility of a foreign body that is ingested and passed down the whole gastrointestinal tract to injure the anorectal mucosa causing the formation of an abscess in the perianal or gluteal area.

## Case presentation

Case 1

A 55-year-old female (height: 167.6 cm and weight: 104.8 kg) with a past medical history of hypertension and diabetes presented to the emergency department with two weeks of worsening left gluteal pain, swelling, and subjective fever. The patient reports that she was previously seen at an outside facility one week ago and given oral clindamycin with minimal improvement. The pain was exacerbated with prolonged sitting. The patient denied any trauma to the area and that she had no symptoms of chills, nausea, or vomiting. The patient also stated that her last colonoscopy was two years ago and it was normal.

On physical exam, the vital signs were within normal limits except for a blood pressure of 160/93 mmHg. On examination of her left gluteal region, a 6x6 cm area of induration, erythema, warmth, and tenderness were observed without fluctuation or crepitus. No tenderness was noted around the perineal region.

**Figure 1:**
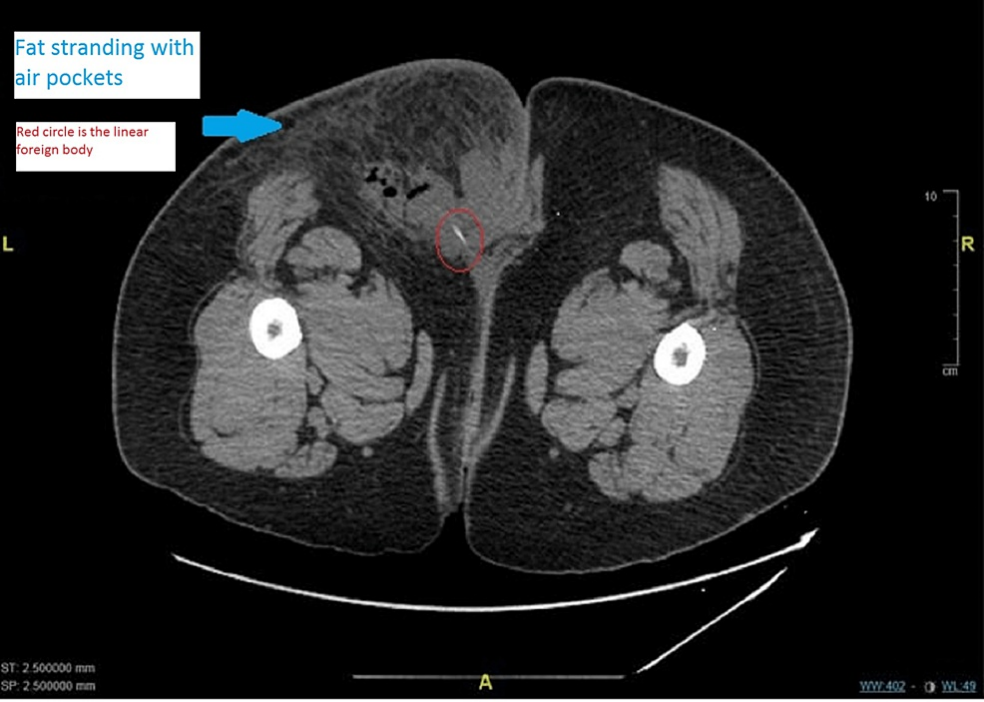
CT scan of pelvis (prone position, axial section) showing linear foreign body (red circle) in left gluteal region abscess with air pockets and subcutaneous fat stranding (blue arrow)

**Figure 2:**
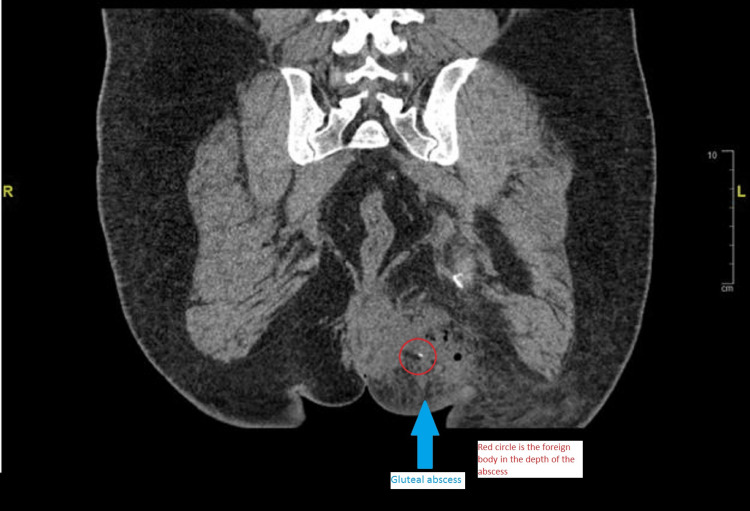
CT scan of pelvis (coronal section) showing linear foreign body (red circle) in left gluteal region abscess with gas pockets and subcutaneous fat stranding (blue arrow)

The patient underwent a computed tomography scan of the pelvis with contrast, which demonstrated a collection of air pockets in the left gluteal region suggestive of an abscess with a needle-like foreign body within the cavity (Figure 1, 2).

**Figure 3:**
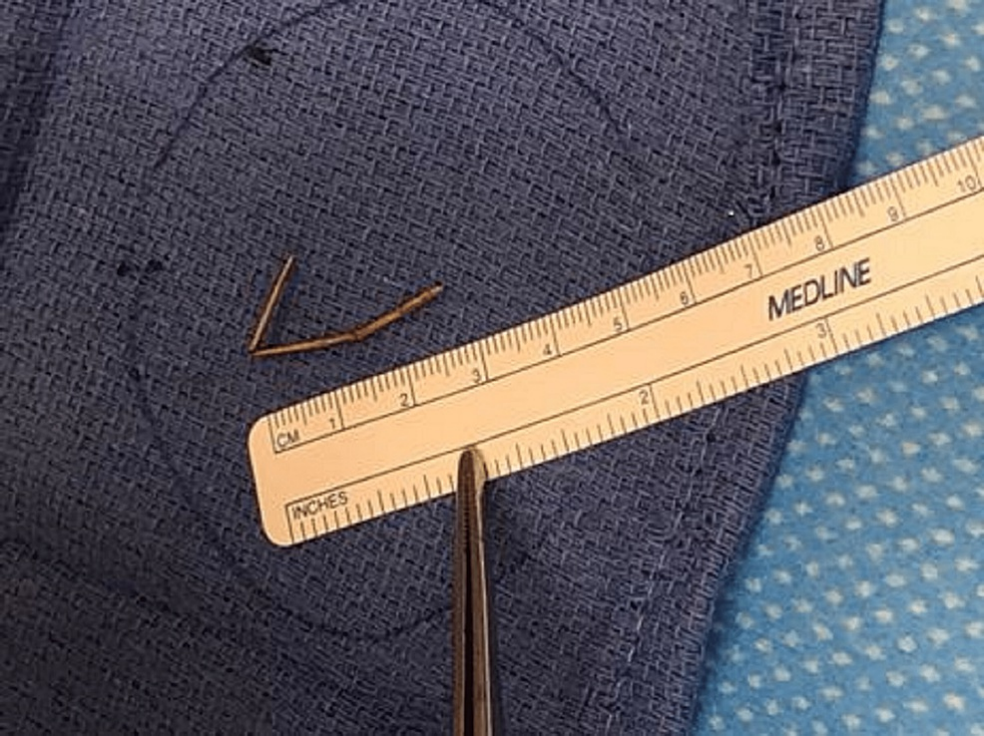
Linear foreign body, a toothpick

The management options were discussed with the patient and she agreed with incision and drainage of the left gluteal abscess under general anesthesia. The patient was in right lateral position, local anesthesia was injected, and incision and drainage were performed over the most fluctuant area. A quantity of 50-75 ml of pus was drained from the abscess cavity, and then it was irrigated with dilute betadine and hydrogen peroxide, and a 3.5 cm long foreign body, a toothpick, was removed from the cavity (Figure 3). The patient did very well after surgery. On further questioning, the patient did not recall swallowing a toothpick and denied any rectal insertions. The patient was discharged home after 48 hours with oral antibiotics and local wound care. No recurrent abscess or fistula was noted in the postoperative follow-up for 18 months.

Case 2

A 59-year-old male patient, with no known past medical history, presented to the emergency department with complaints of perianal pain for the prior one week. His symptoms had worsened during this time. He complained of chills and subjective fever at home and pain while defecating for the past one week. He denied any abdominal pain, nausea, vomiting, or diarrhea. He denied any weight loss or anorexia. He has never had a colonoscopy in the past. He gave a history of symptomatic hemorrhoids. He denied any foreign body insertion in the rectum. On further questioning, the patient mentioned that he ate a significant amount of fish two days earlier.

On physical exam in the emergency room, he had a temperature of 100.3F but the remaining vital signs were normal. His abdomen was soft and non-tender without any guarding or rigidity. Examination of the perianal region showed large induration with fluctuation on the right side. He had a significant leukocytosis of 20,000 with neutrophilia.

**Figure 4:**
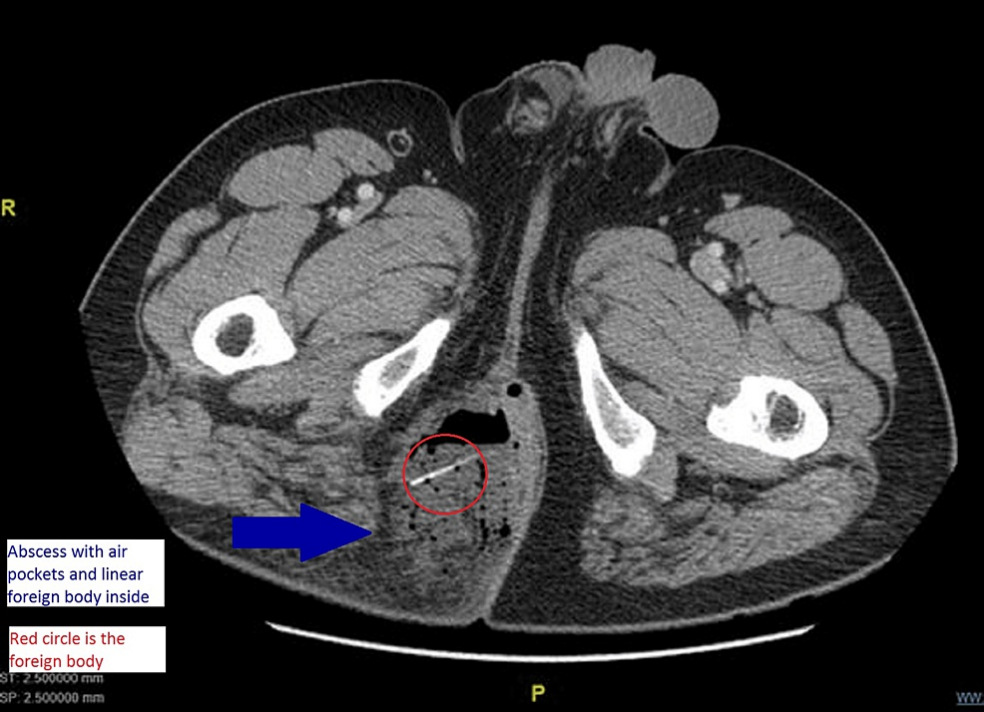
CT scan (axial cut) showing right gluteal abscess with a linear foreign body (red circle)

**Figure 5:**
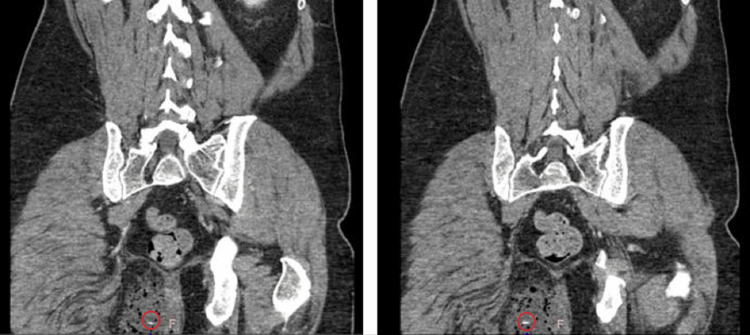
CT scans (consecutive coronal cuts) showing the right gluteal abscess with linear foreign body (red circles)

He underwent a CT scan of the abdomen and pelvis, which showed a large complex fluid collection involving the subcutaneous tissues of the right medial gluteal cleft suggesting a large abscess with a nonspecific linear echogenic structure within the abscess concerning for a linear radiodense foreign body (Figures 4, 5).

The management options were discussed with the patient and he agreed for examination under general anesthesia and incision and drainage of the abscess. The patient was brought to the operating room for examination under anesthesia. The skin over the most fluctuant area was anesthetized with local anesthesia and a 6 cm linear incision was made over the fluctuant area. A quantity of 60 mL of foul-smelling purulent fluid was drained. The cavity was very deep. The loculations were broken with a mix of sharp and blunt dissection. Deep in the perirectal region, there was a palpable foreign body that was removed. The foreign body appeared to be a fishbone measuring around 5 cm. The abscess cavity was irrigated and a Penrose drain was placed.

Postoperatively, the patient did well. The patient was discharged home after 48 hours with oral antibiotics and local wound care. No recurrent abscess or fistula was noted in the postoperative follow-up for 6 months.

## Discussion

Foreign bodies can cause perforations anywhere in the GI tract causing sometimes intra-abdominal abscesses or also forming a fistula, which can range from tracheoesophageal fistulas to colocutaneous fistulas [4,5]. There have been cases where a toothpick caused intestinal perforation and entered the inferior vena cava [6]. In relation to the case we presented, the occurrence of peri-rectal perforation secondary to foreign body ingestion is rare with very few similar cases described in the literature [7,8]. The idea of a pointed foreign body traveling throughout the entire alimentary canal without causing perforation until the rectum is difficult to imagine, as more commonly the foreign body causes small or large bowel perforation as many cases of such have been reported; however, our cases highlight this rare event through the imaging findings.

The results of the CT scan and the operative findings raise the question of how the toothpick or the fishbone ended up in the gluteal region. The patients denied any trauma to the rectal region, including sitting on the toothpick or the fishbone, so the other possibility would be the ingestion of these items and subsequent travel through the gastrointestinal tract resulting in the formation of a gluteal abscess. On the CT scan, the collection of trapped air pockets within the gluteal region but not tracking externally suggests that the foreign body could not have come from an external setting but rather from the alimentary canal.

A suggested mechanism is that during defecation, enough pressure is generated by the evacuating fecal matter to cause the pointed foreign body to perforate through the rectum into the surrounding gluteal region [9]. In previous literature, if a fistulous tract was formed, the common fistulas were rectovaginal and rectovesicular fistulas [5]. Foreign bodies described in fistula formation included bones from various animal products, sutures, and clips [10,11]. The finding of a larger toothpick and also the fishbone coupled with the final location of these foreign bodies were strange in that they missed the closer internal structures and ended up in the gluteal region. Basically, it depends on the direction of perforation, whether for example the foreign body pierces anteriorly causing rectovaginal fistula, or piercing at the side causing an abscess.

In general, the management of these cases includes abscess drainage with foreign body removal. In addition, due to the presence of an abscess and the ‘dirty’ status of the case, incision and drainage was needed to remove the septic source, and antibiotics were added to help with the resolution and prevention of new infection. The laparoscopic approach could also be performed safely instead of open surgery for the removal of ingested foreign bodies when feasible, as in a reported rare case of a foreign body removed at the head of the pancreas laparoscopically [12]; however, minimally invasive would be less feasible in case of perianal or gluteal abscesses like cases described in this report.

In general, it is imperative to say that if a foreign body is suspected, especially as suggested by imaging, then meticulous looking is necessary while incision and drainage are performed to find the foreign body and remove it. Failure to remove the foreign body would cause a considerably high chance of poor healing, recurrent abscess, or further movement of the foreign body elsewhere.

## Conclusions

The presence of a gluteal abscess with a foreign body should raise suspicion of foreign body ingestion with rectal perforation. It happens that foreign bodies can be swallowed either voluntarily or involuntarily but these, whether causing any symptoms or not during passage through the gastrointestinal tract, usually end up coming out with stool during defecation. Although rare, similar cases have been documented in which a swallowed foreign body causes a rectal/gluteal abscess, and removal of the foreign body is required together with draining the abscess cavity.
